# Coronary Spasm in Neurosurgical Patients and Role of Trigeminocardiac Reflex

**DOI:** 10.1155/2014/974930

**Published:** 2014-01-27

**Authors:** Tumul Chowdhury, Cyrill Meuwly, Nora Sandu, Ronald B. Cappellani, Bernhard Schaller

**Affiliations:** ^1^Department of Anesthesia and Perioperative Medicine, Health Sciences Center, University of Manitoba, 671 William Avenue, Winnipeg, Canada R3E 0Z2; ^2^University Hospital of Basel, Switzerland; ^3^Department of Research, University of Southampton, University Road, Southampton S017 1 BJ, UK

## Abstract

*Background.* Coronary artery spasm (CAS) is a rarely reported complication in neurosurgical patients and its main causative mechanism was attributed to vagal mediated responses. However, these may be the unusual manifestations of trigeminal cardiac reflex (TCR) which is a well established brain stem reflex observed in various neurosurgical patients. *Methods and Results.* In this review, we have searched for the case reports/papers related to intraoperative coronary spasm in neurosurgical patients and described the role of TCR in this regard. TCR is a possible mechanism in producing CAS in most of the cases in which stimulation occurred at or near the vicinity of trigeminal nerve. It is likely that TCR mediated coronary spasm may be a physiological mechanism and not related to actual myocardial insult apparent by cardiac enzymes or echocardiography studies in most of the cases. Some common risk factors may also exist related to occurrence of CAS as well as TCR. *Conclusions.* In conclusion, neurosurgical procedures occurring at the vicinity of trigeminal nerve may produce CAS even in previously healthy patients and may produce catastrophic consequences. There is a need for future reports and experimental studies on the interaction of TCR and pathophysiological mechanisms related to CAS.

## 1. Introduction

Hemodynamic disturbances are commonly reported complications in patients undergoing various neurosurgical and skull base interventions [1–3]. These changes have wide array of manifestations including bradycardia, even asystole, with and without hypotension, and may be attributed to various (patho) physiological mechanisms [1–3]. In this regard, central and peripheral trigeminal cardiac reflex (TCR) is a well established neurogenic mechanism which can also cause these changes; however, the role of TCR to produce some unusual cardiac manifestations such coronary artery spasm (CAS) has been rarely reported [2–9].

In this review, we have tried to investigate the possible role of TCR for causing coronary spasms which are unrelated to cardiac changes in different neurosurgical cases previously reported in the literature.

## 2. Method

We did a Pub Med search for the terms “coronary artery spasm,” “neurosurgery”, “intraoperative,” “oculocardiac reflex,” and “Trigeminal-cardiac reflex” from 1 January 1970 to 31 March 2013 for all the relevant publications in any language.

## 3. Coronary Spasm in Neurosurgery

CAS is defined as a transient abnormal contraction of an epicardial coronary artery that results in myocardial ischemia. Usually, there occurs vasospasm at the site of coronary atheroma and commonly linked with endothelial dysfunction [10]. However, the perioperative CAS remains a special concern especially when associated with causes of noncardiogenic origin [10]. In neurosurgical procedures, presence of perioperative CAS is also reported in some cases (Table 1). However, the most common cause for this event has been attributed to vagal stimulation [4–9].

## 4. Exemplary Cases

In one patient (69 y, male) who was undergoing cerebello-pontine tumor operation, CAS was reported during the time of surgical stimulation and presented as sudden marked ST-elevation followed by ventricular fibrillation [4]. In this case, resuscitation was immediately started and surgery was cancelled. The proposed mechanism was presumed to be vagal stimulation [4].

In another case (60 y, female) of a cerebral aneurysm clipping, bradycardia, and atrioventricular block, followed by ST-elevation and ventricular tachycardia, CAS was noted after the surgical incision as well as during the clipping and the cause for CAS was attributed to vagal stimulation and concomitant use of drugs including B-blocker and PG-E1 infusion [5].

CAS has also been reported in small case series (*n* = 2, 70 y and 72 y, both female) in patients undergoing DBS surgery and manifested as chest pain, hypertension (MAP = 113 mm Hg), tachycardia, and ST-changes [11]. These changes were reported during the stimulation of the different thalamic nuclei at the time of electrode insertions [11]. In this report, author did not explain the occurrence of CAS during deep brain stimulation; however, animal experiments suggested the possible association of various cardiovascular changes during different thalamic nuclei and related structures [12, 13].

The other case of CAS was reported in patient (79 y, female) undergoing radiofrequency trigeminal rhizotomy for trigeminal neuralgia under monitored anesthesia care [6]. This patient manifested as chest pain with slight decrease in the heart rate as well as blood pressure but showed marked ST-elevation. These signs were noted during the needle placement through foramen ovale and attributed to vagal response. Activation of the vagal tone reflex may have been induced by stimulation of the dura mater during cannula placement. The cardiac changes were reverted in this case [6]. In another case of glycerol trigeminal rhizotomy (63 y, female), under monitored anesthesia care, patient developed sudden severe hypertension (MAP = 143 mm Hg) and ventricular premature beats followed by transient bradycardia [7]. This patient experienced significant chest discomfort and 12 lead ECG revealed ST-depression which progressed to non-Q wave myocardial infarction (MI). Glycerol injection at Meckel's cave might stimulate the Gasserian ganglion which in turn led to sudden sympathetic charge and produced these cardiovascular changes [7].

Vagal mediated reflex was also thought to attribute to some cases which presented with changes in heart rate, hypotension, ST-elevation, and even ventricular fibrillation during drilling for burr hole [8, 9]. In this case (52 y, male) when burr holes were made for the craniotomy, patient developed bradycardia without changes of MAP suddenly and the ECG in lead II showed sudden elevation of the ST-segment (0.3 mV). After about 1 min, the ST-segment elevation returned to normal and no pharmacological intervention was needed [9]. Interestingly, in one patient (59 y, male) sudden hypotension (MAP = 50 mm Hg) and ventricular fibrillation developed just after the positioning and the probable cause was attributed to induced hyperventilation (PaCO_2_ = 28 mm Hg) for the management of raised intracranial pressure [14].

## 5. Role of Trigeminal Cardiac Reflex

**Figure 1:**
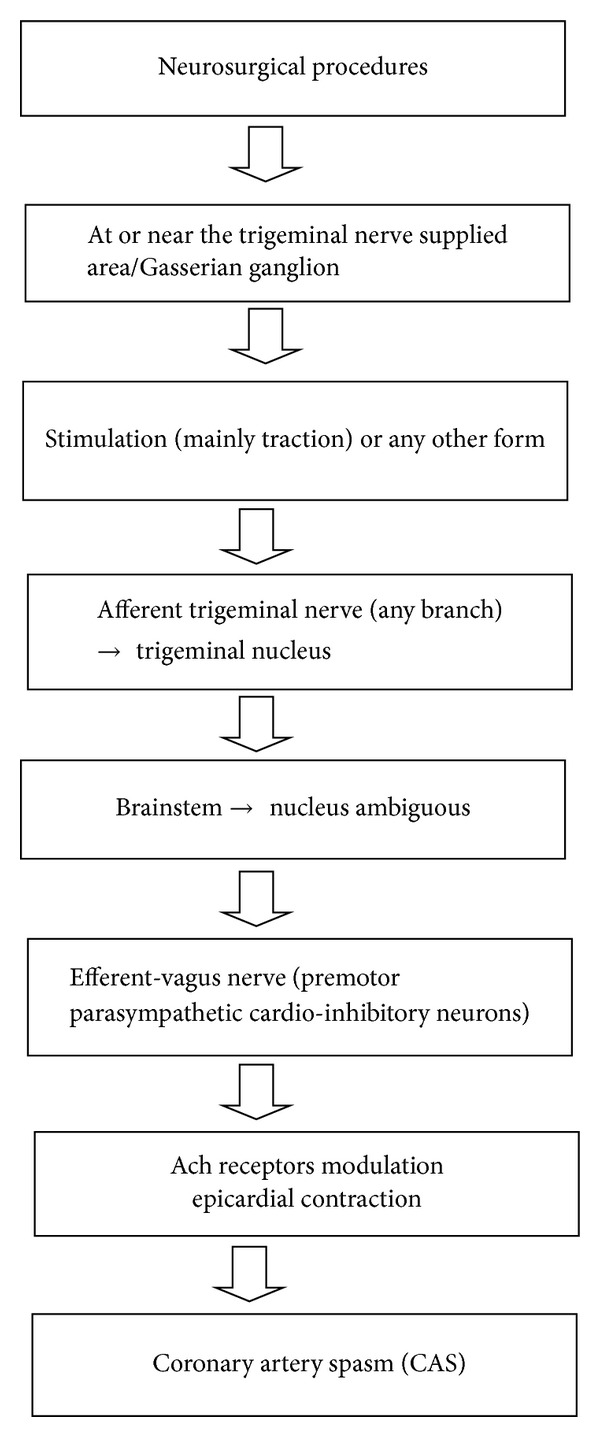
Proposed mechanism of coronary artery spasm evoked by TCR in neurosurgical patients.

The most of the above mentioned neurosurgical cases presented with wide array of cardiovascular changes pertaining to CAS and were attributed to vagal tonic reflexes [4–9]. CAS is the manifestation of autonomic disturbances in most of the cases. The vagal mediated acetylcholine receptors have been also linked with the development of CAS (Figure 1). However, this vagal reflex is a part of TCR which is characterized by hypotension, bradycardia, apnea, and gastric hyper motility [2, 3, 15–18]. This reflex is incited by stimulation of any branch of trigeminal nerve along its course. The exact mechanism is still not fully elucidated; however, it is a part of brain stem reflex which carries signals from trigeminal nerve and relays via vagus nerve. The pathway continues from the ventral trigeminal nucleus through the short internuncial nerve fibers in the reticular formation in the brain stem to finally synapse on efferent premotor parasympathetic cardioinhibitory neurons in the nucleus ambiguous [2, 3]. Therefore, the reported cases in which either the dura was manipulated or surgical stimulation was present on or near to trigeminal nerve vicinity, in fact, incited the TCR and produced the changes such as bradycardia and hypotension. Dura mater is innervated by meningeal branch of maxillary nerve; therefore, stimulation (in the form of stretch) during craniotomy or during burr hole might have provoked TCR and presented with described cardiovascular changes [2, 15–18]. The presence of variable hemodynamics such as hypotension or hypertension caused by TCR was noted during different neurosurgical procedures, probably reflects the central and peripheral pathway of TCR [19, 20]. If there exists complex pathways or subdivisions of trigeminal nerve that lead to CAS it should be further elucidated; however, currently there is no evidence for this. The role of Gasserian ganglion, strength of stimuli, and presence or absence of general anesthesia might play a role in the different occurrences of arterial tension in TCR associated with CAS [2, 19, 20].

In cases of trigeminal rhizotomy, either passage of the needle through the foramen ovale or the stimulation of Gasserian ganglion by glycerol or electric current is usually sufficient to evoke TCR and can manifest as severe hypertension, tachycardia, and other ECG changes. The presence or absence of general anesthesia may interfere with these hemodynamic changes. In addition, the role of Gasserian ganglion is yet to be fully understood; however, some reports suggest that this ganglion is related to sympathetic stimulations and causes autonomic dysfunction. Therefore peripheral TCR usually manifests as hypertension and tachycardia; however, during skull base tumor dissection, central TCR acts which usually produces hypotension and bradycardia [2, 19, 20]. As the trigeminal terminals are found on all vessels of the circle of Willis and their distal branches spread throughout the adventitia [21], the cell bodies of the supratentorial meningeal and cortical vessels lie within the ophthalmic division of the trigeminal ganglion. Therefore reported cardiovascular changes and development of CAS during aneurysm clipping may be possibly contributed to TCR [5, 21, 22].

From the experimental findings, the TCR represents an expression of a central reflex leading to rapid cerebrovascular vasodilatation generated from excitation of oxygen-sensitive neurons in the rostral ventrolateral medulla oblongata [23, 24]. By this physiologic response, the systemic and cerebral circulations may be adjusted in a way that augments cerebral perfusion [23, 24]. Therefore it is likely that TCR mediated coronary spasm may be a cardioprotective mechanism and not related to actual myocardial insult apparent by cardiac enzymes or echocardiography studies in most of the cases. However, the exact molecular mechanism related to development of CAS in neurosurgical patients is still not understood.

## 6. Other Mechanisms and Risk Factors

Intraoperative cardiac rhythm disturbances may occur due to anesthetics, light plane of anesthesia, fluid and electrolyte abnormalities, acid-base disturbances, hypoxemia, hypercarbia, hypothermia, and other neurosurgical causes, especially raised intracranial pressure [1]. Several mechanisms have been suggested to explain the cardiac and cerebral injury, including microvascular spasm and increased levels of circulating catecholamines [1]. In animal study, the arrhythmia-inducing area was found to lie dorsal and caudal to the optic chiasma and to extend caudally in the fornix. Stimulation within the medial dorsal nucleus of the thalamus and the substantia nigra has shown to increase in BP, HR, and RR [12]. On the other hand, stimulation in the caudate nucleus produced either tachypnoe or respiratory arrest accompanied by a slight change in BP and HR. Increase in the arterial blood pressure (BP), heart rate (HR), and respiratory rate (RR) was also evoked by electrical stimulation of the globus pallidus (GP) in an animal model under awake conditions [13]. Therefore, it is likely that stimulation of thalamic nuclei and other structures related to basal ganglia during deep brain stimulation surgery could incite all the described cardiovascular changes including CAS in the reported cases [11–13]. The addition of sympathetic surge produced by thalamic and hypothalamic stimulation may also mimic cardiac ischemia-like changes.

In the perioperative period, CAS has been associated with the recent use of cocaine, hyperventilation, acute withdrawal of beta receptor, and calcium channel blockers and the use of 5-hydroxytryptamine type 3 receptor antagonists as well as sympathomimetic drugs, such as ephedrine [10, 25]. Similarly, some of the provoking factors for the development of CAS are also found to be risk factors for inciting TCR (Table 2). In this, preexisting vagal tone, light-plane of anesthesia, and hyperventilation are worth mentioning [2].

Strikingly, in most of the cases female gender and elderly age group are noted common findings. The female gender has been linked with increased risks for coronary vasospasm due to presence of smaller diameter of coronary artery as well as shorter cardiac cycle, therefore, more prone to manifest cardiac changes in the event of CAS [26, 27]. The protected effect of female hormones against coronary artery disease (CAD) fades after menopause; therefore, this could be one possible explanation for increased incidence of CAS in above discussed cases [26, 27].

## 7. Management

Most of the reported events are of transient nature and persisted for few minutes to few hours. However, few patients developed perioperative myocardial infarction, therefore, need prompt management. There should be thorough preanesthetic checkup to rule out any preexisting ischemic heart disease [10]. In almost all cases, cessation of the stimuli followed by use of nitrates was the first line of treatment [6–8]. Avoidance of hyperventilation and ephedrine use should be ensured and adequate oxygenation should be maintained. Use of B-blocker and CCB are useful for the management of CAS, however, may provoke TCR and worsen or reprecipitate [2, 10, 25]. TCR induces cardiovascular changes, therefore, should be used cautiously. TCR is usually aborted by the cessation of stimuli and sometimes warrants anticholinergic treatment; however, the role of this treatment during CAS is very limited and should be cautiously used. In addition, pacing devices should also be available and rarely warrants cardiopulmonary resuscitation in event of ventricular fibrillation and persistent asystole [4, 8, 10, 14].

## 8. Conclusion

In conclusion, neurosurgical procedures occurring at the vicinity of trigeminal nerve may produce CAS even in previously healthy patients and may produce catastrophic consequences. Therefore understanding of the differential causes including TCR and their prompt management would certainly impart better outcome. There is a need for future reports and experimental studies on the interaction of TCR and pathophysiological mechanisms related to CAS.

## Figures and Tables

**Table 1:** Cases related to coronary artery spasm during neurosurgical procedures.

Author/year	Procedure	Presentation	Treatment
Harada et al. (2011) [4]	CP angle tumor	ST-elevation, VF	Resuscitation/surgery cancelled
Kotake et al. (2009) [5]	Aneurysm clipping	Bradycardia, AV block ST-elevation, VT	Transdermal Isorbide nitrate Stop B-blocker, PG-E1infusion
Glossop and Dobbs (2008) [11]	DBS surgery		
Case 1	Parkinson disease	ST-depression	Sublingual GTN
Case 2	Essential tremors	Tachycardia, HT ST-elevation	Sublingual GTN
Bilgin et al. (2002) [6]	RF trigeminal rhizotomy	Hypotension, bradycardia ST-elevation	IV NTG
Kariya et al. (1999) [8]	Drilling (burr hole)	Hypotension, VF	IV NTG
Furuya et al. (1996) [9]	Burr hole	Bradycardia, ST-elevation	No treatment
Saito et al. (1991) [14]	Craniotomy	Hypotension, VF ST-elevation	Cardiopulmonary resuscitation IV NTG and lidocaine, Surgery cancelled
Swerdlow et al. (1988) [7]	Glycerol trigeminal rhizotomy	ST-changes, MI	Nitrates, B-blocker

CP: Cerebello-pontine; HT: Hypertension; VF: Ventricular fibrillation; VT: Ventricular tachycardia; MI: Myocardial Infarction; NTG: Nitroglycerine.

**Table 2:** Risk factors related to coronary artery spasm and trigeminal-cardiac reflex.

Coronary artery spasm	Trigeminal-cardiac reflex
Common factors	
Hyperventilation	
Hypoxemia	
Light plane of anesthesia	
Preexisting vagal tone	
Sympathomimetics	Opioids
Withdrawn of B-blockers/CCBs	Use of B-blockers/CCBs
Use of suxamethonium	
Hypotension	
Female gender?	
Postmenopausal	

CCB: calcium channel blocker.
